# Fire acupuncture for plaque psoriasis case series

**DOI:** 10.1097/MD.0000000000037848

**Published:** 2024-04-19

**Authors:** Rui-Ming Chen, Guo-Ao Shi, Yong-Qin Xiong, Zhong-Xian Li, Xiang Ji, Yan-Yan Feng, Luda Yan, Xia-Yun Zhou, Hai-Yan Xu, Ting Wu, Shi-Yun Chen, Hai-Fang Gan, Yi-Fu Zhou, Min Peng, Peng Zhou, Jing-Chun Zeng, Jing-Jing Li

**Affiliations:** a Shenzhen Bao’an Traditional Chinese Medicine Hospital, Guangzhou University of Chinese Medicine, Shenzhen, China; b The Clinical Medical College of Acupuncture and Rehabilitation of Guangzhou University of Chinese Medicine, Guangzhou, Guangzhou, China; c The First Affiliated Hospital of Guangzhou University of Chinese Medicine, Baiyun District, Guangzhou, China.

**Keywords:** acupuncture therapy, fire acupuncture, psoriasis

## Abstract

**Objective:**

To investigate the clinical efficacy of fire acupuncture (FA) on plaque psoriasis (PP), exploring its suitable syndrome types, in order to achieve better therapeutic effects, accelerate the possibility of psoriasis skin lesion recovery, and provide assistance for clinical treatment.

**Methods:**

A total of 8 patients with PP aged between 18 and 60 years were recruited and treated with FA once a week, and the lesion area and severity index (PASI), visual analog scale and pruritus were measured before, 2, 4 and 8 weeks after treatment and at the follow-up period (week 12), respectively. Visual analog scale, and dermoscopy were used for assessment.

**Results:**

All patients showed improvement in pruritus after 1 FA treatment, and lesions were reduced to varying degrees after 2 weeks. Except for patients 5 and 8, who only achieved effective results due to severe disease, all other patients with psoriasis achieved significant results at 8 weeks after treatment.

**Conclusion:**

FA can significantly control the development of lesions, reduce the symptoms of PP lesions and pruritus, and help prevent psoriasis recurrence.

## 1. Introduction

Plaque psoriasis (PP) is the most common form of psoriasis among the common types.^[1]^ The clinical manifestation is a dark red plaque covered with silvery white scales, scraping off the scales there are thin films and dewdrop-like bleeding spots. The course of the disease is often long, the lesions are stubborn for a long time, and the recurrence rate is high, which is not easy to cure. The treatment of psoriasis mainly relies on conventional drugs (e.g., A vitamin A, methotrexate, cyclosporine, and biologics) combined with traditional Chinese medicine.^[2]^ Western medicine treats common psoriasis mainly with topical hormone ointment.^[3]^ But it is prone to adverse reactions such as pruritus, burning sensation, stinging sensation, dry skin, erythema and rash.^[4]^ And is poorly tolerated by patients, with a relapse rate of up to 67% within 3 months, and the long-term efficacy has not been proven; for lesions of small size, traditional medicine often focuses on external treatment of which fire acupuncture (FA) is a more advantageous acupuncture measure. FA is a method to treat the disease by burning on the fire until it is white and then stabbing rapidly at the lesion or acupuncture point to the depth of slight bleeding from the lesion.^[5]^ It has been shown that FA can improve the clinical symptoms and lesion area of psoriasis by inhibiting the proliferation of T lymphocytes, and it has also been shown that FA has the effect of anti-oxidative stress and immune regulation, and can achieve the effect of treating skin diseases by promoting the differentiation of regulatory T cells and suppressing the inflammatory response.^[6]^ In this paper, we reported 8 cases of PP treated with FA. Quantitative scoring was performed by erythema, pruritus, infiltration and scaling of lesions and the efficacy was assessed. The study was approved by the Medical Ethics Committee of Shenzhen Baoan District Hospital of Traditional Chinese Medicine (ethical approval number: KY-2022-006-01).

## 2. Methods

### 2.1. Inclusion criteria

Patients with PP diagnosed with lesions covering <50% of the body surface and who had not received systematic treatment or had not used glucocorticoids or retinoids topically in the last month were included in this study. Prior to treatment, we explained to each patient the therapeutic procedure using FA, as well as the safety and precautions to be taken after treatment. We then gave each patient or their legal guardian 30 minutes to decide and obtain verbal consent from all patients or their legal guardians and to sign an informed consent form in the treatment room prior to treatment.

### 2.2. Treatment protocol

We obtained the consent of all patients or their guardians before publication. 75% alcohol was used to sterilize the skin lesions, and all needles were disposable sterile stainless steel acupuncture needles (0.30 × 40 mm from Changchun Aikang Medical Devices Co., Ltd.), and the number of needles (usually 3–5) was selected according to the patient tolerance level and skin lesion area. Using an alcohol lamp for heating, burn the needles until they are white and bright, and quickly puncture the skin lesion vertically, with each needle spaced about 3 to 5 mm apart and the tip of the needle not exceeding the base of the skin lesion. After the fire needle, gently wipe with a cotton swab dipped in alcohol. The wound was instructed to be kept free of water for 24 hours. Fire needling treatment was performed once every 1 week, and lesion repair was observed every 2 weeks using a dermatoscope. At 2 weeks after the initial treatment, the patient condition was evaluated. If the FA treatment is effective and there are no new lesions, the patient can be treated with FA again. If adverse reactions such as burns, infections, or homozygous reactions occur during treatment, treatment should be stopped and symptomatic treatment should be given accordingly.

### 2.3. Observation index

Baseline data were recorded in detail during the patient visit. The lesion area and severity index (PASI) was assessed before treatment, at 2 weeks of treatment, 4 weeks of treatment, 8 weeks of treatment, and at the follow-up period (week 12), respectively, and the observed indicators included lesion area, erythema, papules, scaling, and infiltration, using a manual scoring method, with higher scores indicating more severe lesions.^[7]^ Pruritus scores were referenced to the visual analog scale for pruritus, with a range of 0 to 10, with no sensation being 0, mild itchiness being 1 to 3, moderate itchiness being 4 to 7, and severe itchiness being 7 to 10, with higher scores indicating more severe pruritus.^[6]^ After treatment, the efficacy was assessed by the nimodipine method according to the PASI scale: a PASI score of >90% was considered clinically cured, a PASI score of 60% to 89% was considered effective, 30% ≤ PASI score ≤ 59% was considered effective; a PASI score < 30% was considered ineffective.^[8]^

## 3. Results

**Figure 1.**
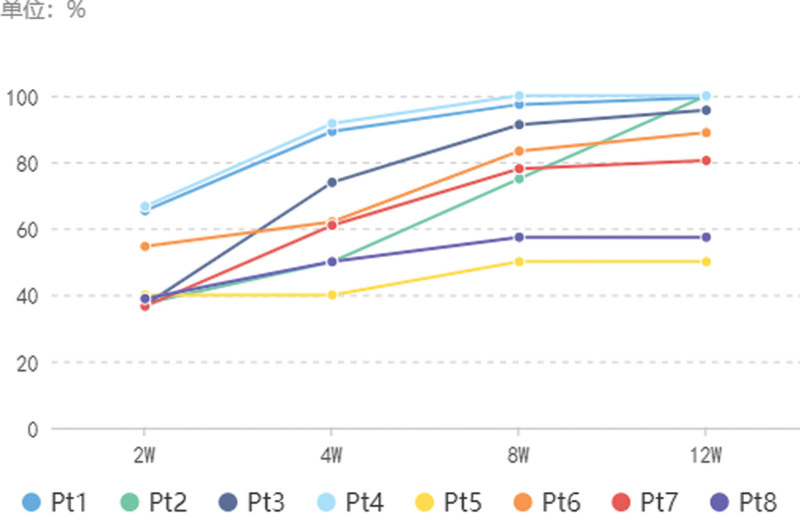
Prognosis of patients in this study. Nimodipine method was used to assess the efficacy according to the PASI scale: PASI score > 90% was considered clinically cured, PASI score of 60% to 89% was considered effective, 25% ≤ PASI score ≤ 59% was considered effective; PASI score < 25% was considered ineffective. PASI = lesion area and severity index.

**Figure 2.**
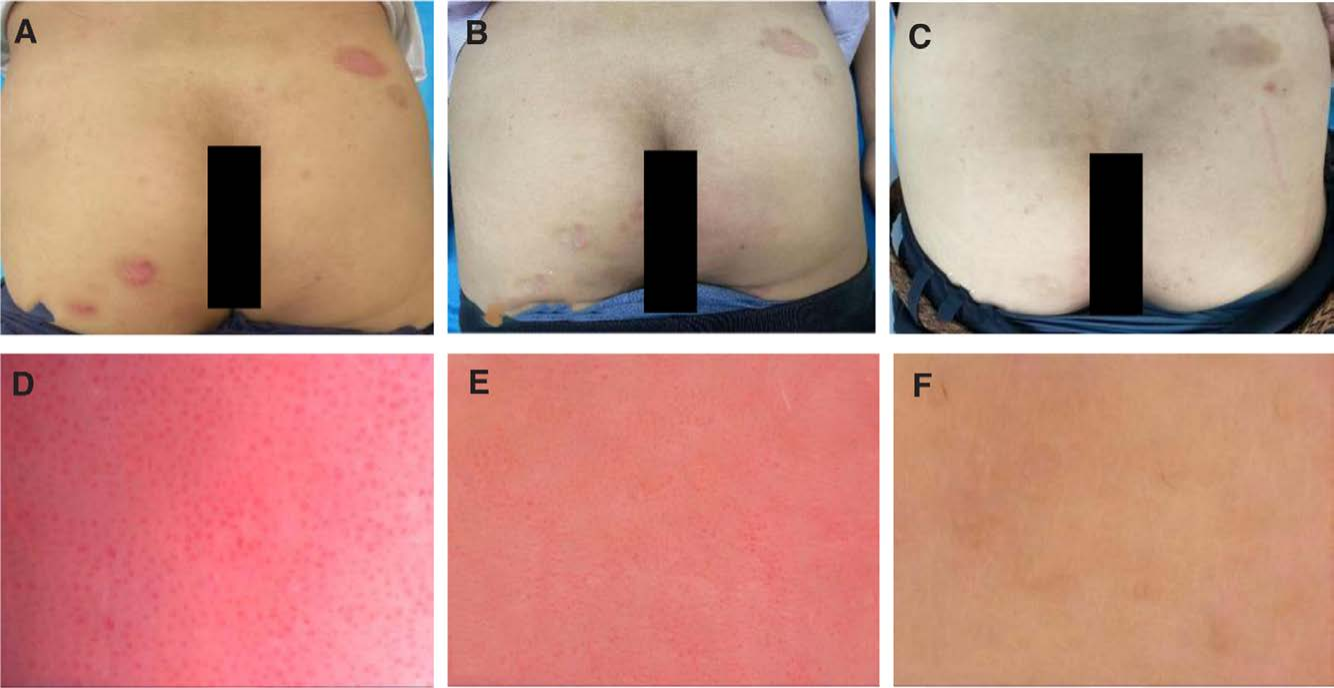
Comparison of skin lesions before and after Pt7 treatment and follow-up period, as well as dermatoscopic findings. (A–C) Pretreatment, post-treatment and follow-up lesions on the buttocks of Pt 7; (D–F) Pretreatment, post-treatment and follow-up dermoscopic manifestations on the anterior chest of Pt 7.

**Figure 3.**
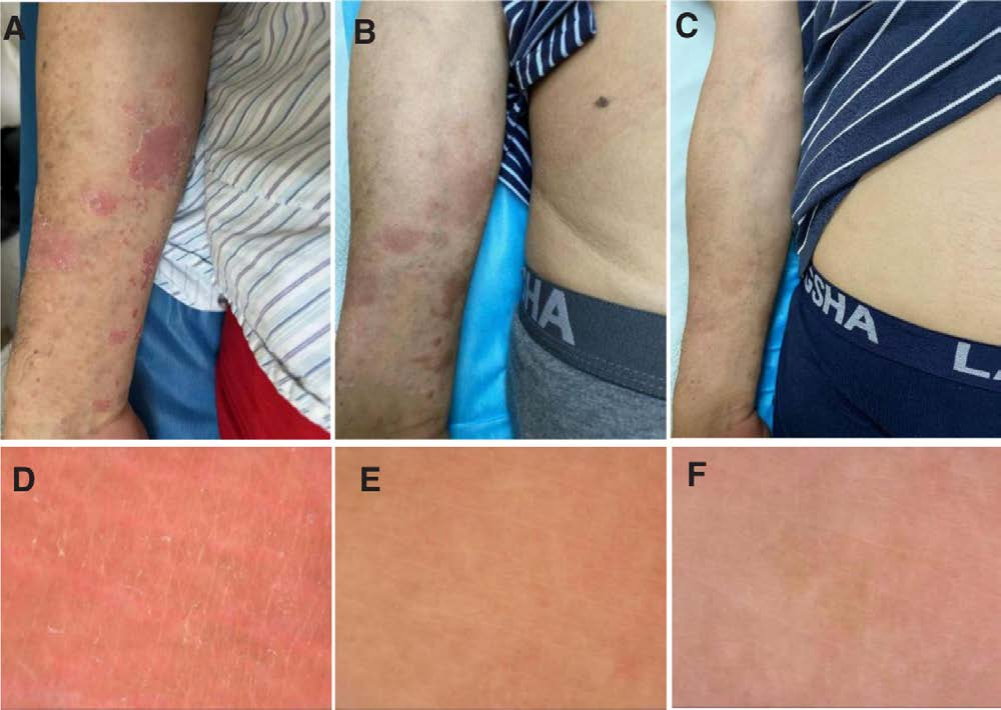
Comparison of skin lesions before and after Pt 8 treatment and follow-up period, as well as dermatoscopic findings. (A–C) Pretreatment, post-treatment and follow-up lesions on the medial forearm of Pt 8; (D–F) Dermoscopic manifestations on the medial forearm of Pt 8 before, after and during the follow-up period.

Our study included 8 patients, 2 women and 6 men, aged between 18 and 60 years. The duration of the disease ranged from 2 to 156 months (Table 1). Patients 3, 4 and 7 did not receive any treatment prior to their first visit. The effect of topical carbotriol ointment was not satisfactory in other patients prior to their first visit. All patients showed improvement in pruritus after 1 FA treatment, and all lesions were reduced to varying degrees after 2 weeks. Patients 2 and 5 had lesions mainly on the head and had relatively poor efficacy and slow recovery. Patient 5 had little change after 4 treatments, and other patients also had deviations in the efficacy of the head. Patients 4 and 7 had lighter lesions and improved significantly after 2 weeks of treatment, with some parts of the lesions recovering close to normal skin, and no recurrence was seen during the follow-up period. Patients 1, 6, and 8 had larger lesions and longer disease duration and each received 2, 2, and 4 additional sessions of FA treatment, achieving significant and effective results at 10 and 12 weeks after treatment, respectively. Except for patients 5 and 8, who were only effective due to their severe disease, all other patients with psoriasis achieved significant effect at 8 weeks after treatment (Table 2 and Fig. 1). Representative images are shown in Figures 2 and 3. All treatments were well tolerated, with patients complaining of moderate pain at most, which subsided rapidly after the fire needle intervention. We did not find any other side effects except for needle-induced pain. The research results indicate that after fire needle treatment, the PASI score of all patients significantly decreased, and the background, scales, and vascular types of local skin lesions under the microscope improved to varying degrees. This indicates that fire needle treatment can significantly improve symptoms, reduce the economic burden and time consumption caused by long-term medication and topical use, and improve the quality of life of patients. However, patients with long course, generalized, head and facial, and infiltrative skin lesions may have slower recovery and may still require comprehensive treatment. In terms of limitations, the symptoms of PP are complex and diverse, and some included cases lack unified evaluation criteria and quantitative treatment plans for efficacy evaluation; Lack of large sample, multicenter randomized controlled studies; Insufficient attention to follow-up in the later stage, unable to accurately evaluate its recurrence rate, thus affecting its efficacy evaluation.

**Table 1:** Baseline data of this study (by VAS, PASI scores).

NO	Age	Gender	Duration (mo)	Baseline (before treatment)					Number of treatments
				Area	Symptoms				
					Pruritus	Scaling	Infiltration	Erythema	
Pt 1	42	M	156	HF (2) UE (3) T (3) LE (5)	HF (5) UE (5) T (5) LE (5)	HF (4) UE (4) T (4) LE (4)	HF (2) UE (1) T (2) LE (1)	HF (4) UE (4) T (4) LE (4)	10
Pt 2	41	M	12	HF (1)	HF (4)	HF (3)	HF (2)	HF (3)	4
Pt 3	46	F	62	HF (2) UE (2) T (3) LE (2)	HF (5) UE (5) T (7) LE (6)	HF (3) UE (3) T (3) LE (3)	HF (4) UE (4) T (4) LE (4)	HF (1) UE (1) T (1) LE (1)	6
Pt 4	58	M	8	LE (3)	LE (3)	LE (4)	LE (1)	LE (3)	3
Pt 5	39	F	36	HF (1)	HF (4)	HF (3)	HF (3)	HF (1)	8
Pt 6	58	M	120	T (2) LE (3)	T (3) LE (3)	T (2) LE (2)	T (2) LE (2)	T (2) LE (2)	10
Pt 7	48	M	2	HF (1) T (1) LE (1)	HF (2) T (2) LE (2)	HF (1) T (2) LE (1)	HF (1) T (2) LE (2)	HF (1) T (2) LE (2)	8
Pt 8	60	M	96	HF (5) UE (3) T (3) LE (3)	HF (6) UE (6) T (6) LE (6)	HF (3) UE (3) T (3) LE (3)	HF (4) UE (4) T (2) LE (4)	HF (2) UE (2) T (2) LE (2)	12

Trunk abbreviated as T, head and face abbreviated as HF, upper extremities abbreviated as UE, lower extremities abbreviated as LE.

PASI = lesion area and severity index, VAS = visual analog scale.

**Table 2:** Validity of the study.

No	2 wk		4 wk		8 wk		Follow-up	
	Area	Symptoms	Area	Symptoms	Area	Symptoms	Area	Symptoms
Pt 1	HF (2) UE (2) T (3) LE (3)	Pruritus (4) Scaling: HF (2) UE (3) T (2) LE (2) Infiltration: HF (1) UE (0) T (1) LE (0) Erythema: HF (2) UE (1) T (2) LE (2)	HF (1) UE (1) T (1) LE (2)	Pruritus (3) Scaling: HF (1) UE (1) T (0) LE (1) Infiltration: HF (1) UE (0) T (1) LE (0) Erythema: HF (1) UE (1) T (1) LE (2)	HF (1) UE (0) T (1) LE (1)	Pruritus (2) Scaling: HF (1) UE (0) T (0) LE (0) Infiltration: HF (0) UE (0) T (0) LE (0) Erythema: HF (1) UE (0) T (1) LE (1)	HF (1) UE (0) T (0) LE (0)	Pruritus (0) Scaling: HF (1) UE (0) T (0) LE (0) Infiltration: HF (0) UE (0) T (0) LE (0) Erythema: HF (1) UE (0) T (0) LE (0)
Pt 2	HF (1)	Pruritus (3) Scaling: HF (2) Infiltration: HF (2) Erythema: HF (1)	HF (1)	Pruritus (2) Scaling: HF (2) Infiltration: HF (1) Erythema: HF (1)	HF (1)	Pruritus (1) Scaling: HF (1) Infiltration: HF (1) Erythema: HF (0)	HF (0)	Pruritus (0) Scaling: HF (0) Infiltration: HF (0) Erythema: HF (0)
Pt 3	HF (2) UE (2) T (2) LE (2)	Pruritus (4) Scaling: HF (2) UE (2) T (2) LE (2) Infiltration: HF (4) UE (3) T (3) LE (3) Erythema: HF (1) UE (1) T (0) LE (1)	HF (2) UE (1) T (1) LE (1)	Pruritus (3) Scaling: HF (2) UE (1) T (2) LE (2) Infiltration: HF (3) UE (2) T (2) LE (2) Erythema: HF (1) UE (1) T (0) LE (0)	HF (1) UE (1) T (1) LE (1)	Pruritus (2) Scaling: HF (1) UE (0) T (1) LE (0) Infiltration: HF (2) UE (1) T (1) LE (1) Erythema: HF (1) UE (0) T (0) LE (0)	HF (1) UE (0) T (1) LE (0)	Pruritus (0) Scaling: HF (1) UE (0) T (1) LE (0) Infiltration: HF (1) UE (0) T (1) LE (0) Erythema: HF (0) UE (0) T (0) LE (0)
Pt 4	LE (2)	Pruritus (2) Scaling: LE (2) Infiltration: LE (1) Erythema: LE (1)	LE (1)	Pruritus (1) Scaling: LE (1) Infiltration: LE (0) Erythema: LE (1)	LE (0)	Pruritus (0) Scaling: LE (0) Infiltration: LE (0) Erythema: LE (0)	LE (0)	Pruritus (0) Scaling: LE (0) Infiltration: LE (0) Erythema: LE (0)
Pt 5	HF (1)	Pruritus (3) Scaling: HF (2) Infiltration: HF (2) Erythema: HF (2)	HF (1)	Pruritus (3) Scaling: HF (2) Infiltration: HF (2) Erythema: HF (2)	HF (1)	Pruritus (3) Scaling: HF (2) Infiltration: HF (2) Erythema: HF (1)	HF (1)	Pruritus (3) Scaling: HF (2) Infiltration: HF (2) Erythema: HF (1)
Pt 6	T (1) LE (2)	Pruritus (2) Scaling: T (1) LE (2) Infiltration: T (1) LE (2) Erythema: T (1) LE (1)	T (1) LE (2)	Pruritus (1) Scaling: T (1) LE (1) Infiltration: T (1) LE (2) Erythema: T (1) LE (1)	T (1) LE (1)	Pruritus (0) Scaling: T (1) LE (1) Infiltration: T (1) LE (1) Erythema: T (0) LE (1)	T (0) LE (1)	Pruritus (0) Scaling: T (0) LE (1) Infiltration: T (0) LE (1) Erythema: T (0) LE (1)
Pt 7	HF (1) T (1) LE (1)	Pruritus (1) Scaling: HF (1) T (2) LE (0) Infiltration: HF (1) T (2) LE (1) Erythema: HF (1) T (1) LE (1)	HF (1) T (1) LE (1)	Pruritus (1) Scaling: HF (1) T (1) LE (0) Infiltration: HF (1) T (1) LE (1) Erythema: HF (1) T (1) LE (0)	HF (1) T (1) LE (0)	Pruritus (0) Scaling: HF (1) T (0) LE (0) Infiltration: HF (1) T (1) LE (0) Erythema: HF (1) T (1) LE (0)	HF (1) T (1) LE (0)	Pruritus (0) Scaling: HF (1) T (0) LE (0) Infiltration: HF (1) T (1) LE (0) Erythema: HF (0) T (1) LE (0)
Pt 8	HF (5) UE (2) T (2) LE (2)	Pruritus (5) Scaling: HF (3) UE (2) T (2) LE (2) Infiltration: HF (4) UE (3) T (2) LE (3) Erythema: HF (2) UE (2) T (2) LE (2)	HF (5) UE (1) T (2) LE (2)	Pruritus (4) Scaling: HF (3) UE (1) T (2) LE (2) Infiltration: HF (4) UE (1) T (2) LE (2) Erythema: HF (2) UE (1) T (2) LE (2)	HF (5) UE (1) T (2) LE (2)	Pruritus (2) Scaling: HF (3) UE (1) T (1) LE (2) Infiltration: HF (4) UE (1) T (2) LE (2) Erythema: HF (2) UE (1) T (1) LE (1)	HF (5) UE (1) T (2) LE (2)	Pruritus (2) Scaling: HF (3) UE (1) T (1) LE (2) Infiltration: HF (4) UE (1) T (2) LE (2) Erythema: HF (2) UE (1) T (1) LE (1)

Trunk abbreviated as T, head and face abbreviated as HF, upper extremities abbreviated as UE, lower extremities abbreviated as LE.

## 4. Typical Case Report

A 44-year-old male with psoriasis (Pt 1) was diagnosed with psoriasis in 2009 and treated with oral methotrexate tablets, topical tacrolimus, and phototherapy, but his symptoms recurred over a 12-year period. on December 06, 2021, he had a generalized outbreak of scaly erythema, flaking, and pruritus. On examination, we saw large, well-defined red plaques of varying sizes over the patient entire body, covered with multiple layers of easily peelable gray-white scales, with shiny, pale red films and punctate hemorrhages visible beneath the scales (Fig. 2). His head lesions had thick scales on the surface and his hair was in tufts. Dermatoscopic examination showed a consistent distribution of punctate or globular vessels at low magnification, ring-like or hairpin-like vessels at high magnification, and diffuse distribution of white scales. Weekly FA treatment was used between December 06, 2021 and January 20, 2022. Patients were treated by an attending physician (7th Clinical School of Guangzhou University of Traditional Chinese Medicine) with more than 5 years of clinical experience in clinical acupuncture. The FA site was localized to the skin lesion.

**Figure 4.**
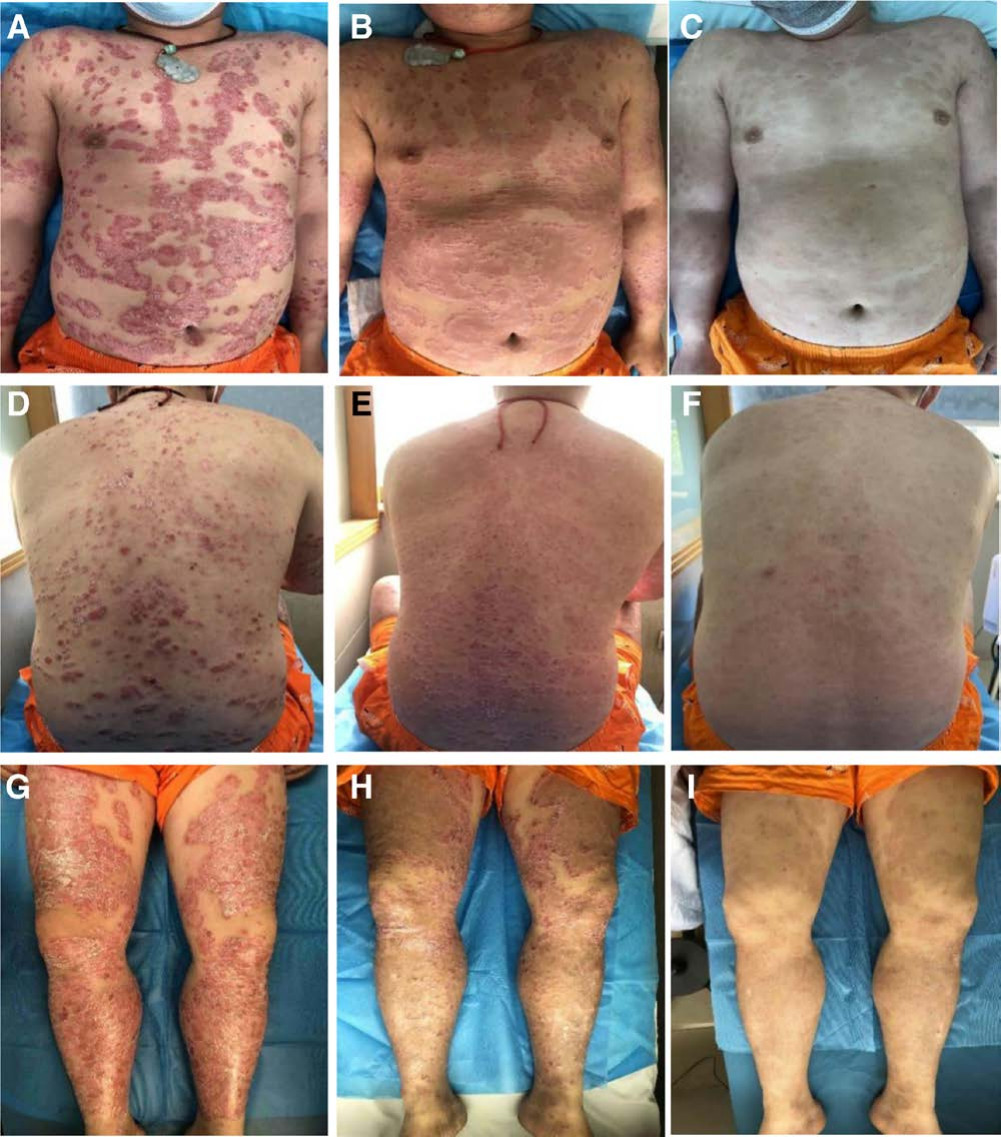
Comparison of skin lesions before and after Pt 1 treatment and follow-up period, as well as dermatoscopic findings. (A–C) Pretreatment, post-treatment, and follow-up lesions of Pt 1 back; (D–F) pretreatment, post-treatment, and follow-up lesions of patient 1 anterior chest; (G–I) pretreatment, post-treatment, and follow-up lesions of Pt 1 bilateral lower extremities.

Procedure: The fire needle is burned in the external flame of an alcohol lamp until it appears white. The lesion area was punctured with a fire needle (FA). On December 27, 2021, the patient local erythema became lighter in color and flattened in size, with reduced surface scaling and no local itching. on April 12, 2022, no local scaling erythema was seen on the patient trunk and extremities, and only A small amount of hyperpigmentation remained. Figure 4 shows a comparison of before and after treatment. During the treatment, the patient did not report any adverse reactions, was satisfied with the FA treatment, and had positive efficacy. At the telephone follow-up after 6 months, the patient reported no recurrence of psoriasis.

## 5. Discussion

Psoriasis is an autoimmune systemic disease in which the immune response is dominant.^[9]^ It is characterized by epidermal hyperkeratosis, leukocyte infiltration, and dermal capillary dilation.^[10]^ The incidence of psoriasis can be as high as 4.8% and can occur at any age, with the 2 peak periods being 15 to 20 and 55 to 60 years of age.^[11]^ 80% of patients with psoriasis have a common type of plaque lesion, which is particularly intractable and is the more severe type of common psoriasis.^[12,13]^ According to the progression, it can be divided into progressive, quiescent, and receding phases, and PP lesions are mostly in the quiescent phase, with dark plaques, clear borders, infiltrative hypertrophy, and a long disease course. Histopathologically, hypertrophy of the spinous layer, epidermal prominence extension, and capillaries curved into a spherical shape can be observed in the lesions.^[14]^ Current mainstream treatment regimens are based on corticosteroids, vitamin A acid, calcium-regulated phosphatase inhibitors, vitamin D analogs, and combination agents, which can effectively inhibit keratinocyte proliferation and induce their differentiation.^[15,16]^ Treatment with biological agents such as etanercept, infliximab, ustekinumab, and secukinumab still has the disadvantages of uncertain long-term efficacy, long treatment course, high price, and high recurrence rate.^[17]^

In the present study, we found that all psoriatic lesions began to show varying degrees of reduction 2 weeks after FA treatment. Among them, erythema, desquamation, and non-infiltrative lesions could be significantly improved in 4 to 8 weeks, and the efficacy could be achieved after 4 weeks of treatment; all patients’ pruritus symptoms could be significantly improved after 1 FA treatment. However, the infiltrative, generalized, lesions located on the head and face improved more slowly, and the overall efficacy was effective after a treatment period of more than 8 weeks. It can be seen that the duration of the disease and the efficacy of treatment show a certain positive correlation. That is, the longer the duration of treatment, the longer the time required for treatment. In this regard, we believe that there may be a link between the type of disease, the duration of the disease and the characteristics of FA. That is, patients with long disease duration, generalized, cephalofacial and infiltrative lesions may still require a combination of treatments. In the future, it remains to be explored the reasons for the poorer remission effect, with a view to achieving better treatment results, accelerating the possibility of healing of psoriatic lesions, and providing a real and objective basis for clinical treatment.

The lesion condition and pruritus of the above 8 patients improved significantly after receiving FA treatment, and all patients in this study were able to live and work normally after receiving Lingnan FA treatment; it was found that after 8 weeks of treatment, patients’ PASI scores decreased significantly compared with those before treatment, with patients 5 and 8 decreasing between 50% and 60%; patients 2, 6 and 7 decreasing between 60% and 90%, patients 1, 3 and 4 decreasing between 90% and 100%, and all patients had a decrease in PASI scores between 50% and 100%. During the period the group used dermoscopy for before and after comparative observation to assist in the diagnosis and assessment of psoriasis patients, which has high application in the differential diagnosis and efficacy assessment of non-pigmented dermatoses, especially erythematous scaly dermatoses, and uniform and consistent red background, scales and densely distributed punctate blood vessels are important dermoscopic features of psoriasis.^[18,19]^ Through clinical observation, we found that the background, scaling, and vessel type of dermoscopic manifestations improved to different degrees in each patient after treatment.

FA reduces the economic burden and time consumption of patients due to long-term medication and topical medication, reduces adverse drug reactions, can significantly improve symptoms, and enhances patients’ quality of life.^[20,21]^ FA has the characteristics of direct access to the disease, rapid effect, high safety, and easy operation.^[22]^ Therefore, we advocate the combination of drug and FA protocols for the treatment of PP. In previous applications, coarse fire needles were used, whereas in this case, a special fine fire needle was used, which can be adjusted according to the area and morphology of the lesion and is easy to operate.

The skin microcirculation and nail fold microcirculation of psoriasis patients have different degrees of impairment.^[23]^ FA has the dual effect of acupuncture and moxibustion, and FA can act on lesions locally through high temperature, lesion tissues are burned to charring, blood flow rate of nail fold microcirculation is significantly accelerated, local microcirculation is improved, and the disease recurrence rate is reduced.^[24]^ It has been shown that the occurrence of psoriasis is related to the imbalance of redox status in patients, and oxidative stress affects the cellular immune function of the body and participates in the development of psoriasis and aggravates the inflammatory response of patients.^[25]^ Dysfunctional helper T cells (Th1, Th17, Th22, and Treg cells) are integral to the development of psoriasis.^[26]^ Fire needling reduces serum levels of globular C1q receptor gene (g C1q R), effector T-cell (Teff) subsets, inhibits oxidative stress indicators such as SOD, LPO, and MDA to reduce psoriasis-causing factor levels, improves cellular antioxidant damage, and regulates the autoimmune system;^[27]^ The IL-23/IL-17 axis plays a central role in psoriasis, and TNF-α, IL-1β, IL-17A, IFN-γ,IL-6, IL-22, IL-26, IL-29, IL-36,etc are inflammatory factors that play an important role in the pathogenesis of psoriasis.^[28,29]^ FA treatment reduces epidermal thickness, inhibits keratinocyte proliferation, and decreases CD3 + T-cell infiltration, while decreasing IL-17A, IL-1β, IL23p40,^[30]^ and IL-22levels in psoriasis local lesions.^[31]^ It has also been reported in the relevant literature that IgE levels in the serum of patients with psoriasis are elevated to varying degrees,^[32]^ which is a typical marker of Th2 immune response,^[33]^ and FA can play a role in adjusting humoral immunity and improving anti-allergic ability by reducing the abnormally high serum IgE levels and inhibiting the release of allergic mediators, which can help improve the pruritus of psoriatic lesions.^[34]^

In this study, the efficacy of fire for psoriasis with predominantly erythematous and scaly symptoms was significant, with a significant reduction in PASI scores in the cases and no relapse during the follow-up period. However, the following problems still exist: the symptoms of PP are complex and diverse, and the efficacy evaluation criteria of some included cases lack unified assessment criteria and quantitative treatment protocols; for PP, there is still a lack of large sample, multicenter randomized controlled studies. Insufficient attention has been paid to late follow-up, which cannot accurately assess its recurrence rate, thus affecting its efficacy evaluation. Therefore, future research on the treatment of psoriasis with FA should focus on the following aspects: increasing the sample size of the study, extending the observation and follow-up time, exploring its appropriate evidence types, and conducting in-depth mechanistic studies to verify the therapeutic principles of FA for psoriasis to provide experimental evidence and theoretical support for clinical practice.

## 6. Conclusion

The aim of this study was to determine the effect of FA on PP. The results of the study showed that FA treatment is a worthy clinical reference because it can effectively improve patients’ symptoms of skin lesions and itching, while relieving anxiety and improving quality of life, and is safe without serious adverse effects, based on low cost and easy operation.

## Author contributions

**Conceptualization:** Jing-Jing Li.

**Data curation:** Zhong-Xian Li, Hai-Yan Xu, Peng Zhou.

**Formal analysis:** Xiang Ji, Ting Wu, Shi-Yun Chen, Hai-Fang Gan, Yi-Fu Zhou, Min Peng.

**Investigation:** Luda Yan.

**Methodology:** Yan-Yan Feng.

**Project administration:** Jing-Jing Li.

**Supervision:** Peng Zhou, Jing-Chun Zeng, Jing-Jing Li.

**Visualization:** Xia-Yun Zhou.

**Writing – original draft:** Rui-Ming Chen.

**Writing – review & editing:** Guo-Ao Shi, Yong-Qin Xiong.
